# A pharmaco-economic analysis of second-line treatment with imatinib or sunitinib in patients with advanced gastrointestinal stromal tumours

**DOI:** 10.1038/sj.bjc.6604367

**Published:** 2008-05-27

**Authors:** I Contreras-Hernández, J F Mould-Quevedo, A Silva, G Salinas-Escudero, M A Villasís-Keever, V Granados-García, G Dávila-Loaiza, J A Petersen, J Garduño-Espinosa

**Affiliations:** 1 Unidad de Investigación en Economía de la Salud, Instituto Mexicano del Seguro Social, Mexico City, MX, Mexico; 2 Department of Pharmaco-economics, Pfizer Pharmaceuticals, Mexico City, MX, Mexico; 3 Oncología Médica, Hospital de Oncología Centro Médico Nacional Siglo XXI, Instituto Mexicano del Seguro Social, Mexico City, MX, Mexico; 4 Unidad de Investigación en Epidemiología Clínica, Hospital de Pediatría, Centro Médico Nacional Siglo XXI, Instituto Mexicano del Seguro Social, Mexico City, MX, Mexico; 5 Pfizer Pharmaceuticals, Global Outcomes Research, New York City, NY, USA

**Keywords:** sunitinib, imatinib, gastrointestinal stromal tumours, markov models, cost-effectiveness analysis

## Abstract

Second-line treatments recommended by the National Cancer Center Network to manage advanced-stage gastrointestinal stromal tumours (GIST) were evaluated to determine the cost and cost-effectiveness of each intervention in the Mexican insurance system, the Instituto Mexicano del Seguro Social (IMSS). Treatments examined over a 5-year temporal horizon to estimate long-term costs included 800 mg day^−1^ of imatinib mesylate, 50 mg day^−1^ of sunitinib malate (administered in a 4 week on/2 week rest schedule), and palliative care. The mean cost (MC), cost-effectiveness, and benefit of each intervention were compared to determine the best GIST treatment from the institutional perspective of the IMSS. As sunitinib was not reimbursed at the time of the study, a Markov model and sensitivity analysis were conducted to predict the MC and likelihood of reimbursement. Patients taking 800 mg day^−1^ of imatinib had the highest MC (±s.d.) of treatment at $35 225.61 USD (±1253.65 USD); while sunitinib incurred a median MC of $17 805.87 USD (±694.83 USD); and palliative care had the least MC over treatment duration as the cost was $2071.86 USD (±472.88 USD). In comparison to palliative care, sunitinib is cost-effective for 38.9% of patients; however, sunitinib delivered the greatest survival benefit as 5.64 progression-free months (PFM) and 1.4 life-years gained (LYG) were obtained in the economic model. Conversely, patients on imatinib and palliative care saw a lower PFM of 5.28 months and 2.58 months and also fewer LYG (only 1.31 and 1.08 years, respectively). Therefore, economic modeling predicts that reimbursing sunitinib over high dose imatinib in the second-line GIST indication would deliver cost savings to the IMSS and greater survival benefits to patients.

Gastrointestinal stromal tumours (GISTs) are rare in the Mexican population (Alvarado-Cabrero *et al*, 2007); nonetheless, this tumour type is expensive to treat as many patients are diagnosed in the advanced and thus more costly stages of disease. Multiple treatment options for second-line GIST exist; yet interventions have not been investigated from the cost and cost-effectiveness perspective of the major payer for oncology treatment in Mexico, the Instituto Mexicano del Seguro Social (IMSS). This is a new predicament as prior to 2001 the only treatment for GIST beside surgical resection was palliative care (Dematteo *et al*, 2002). In fact, no intervention for patients with advanced non-resectable tumours or disease progression had been identified (Emory *et al*, 1999).

However, the discovery that abnormalities in the KIT tyrosine kinase receptor had impact on tumour development led to the creation of targeted GIST therapies (Dematteo *et al*, 2002; Fletcher *et al*, 2002; Hassan *et al*, 2006; Hirota and Isozaki, 2006; Miettinen *et al*, 2006; Hornick and Fletcher, 2007). In 2004, imatinib was approved for use as a first-line treatment for GIST after phase II and III clinical trials yielded overall response rates of approximately 50%, a 1-year survival rate greater than 80%, and a 2-year survival rate around 70% (Joensuu *et al*, 2001; Dagher *et al*, 2002; Demetri *et al*, 2002, 2005; Verweij *et al*, 2004). While imatinib is a promising treatment option for many GIST patients other treatments need to be evaluated for reimbursement as 12–14% of patients show primary resistance; 40% develop secondary resistance after 25 months; and an additional 5% of patients become imatinib intolerant and discontinue therapy (Blanke and Corless, 2005; Van Glabbeke *et al*, 2005). Since the 400 mg dose of imatinib is ineffective in a growing number of advanced GIST patients, alternative therapy options are important in GIST treatment and disease management.

As the cost and cost-effectiveness of second-line GIST treatment has not been thoroughly investigated; reimbursement agencies like the IMSS do not have cost and benefit data specific to different GIST therapies. Given the growing cost of therapy and the increasing dependence on the IMSS to cover treatment, a pharmaco-economic analysis was conducted to determine which therapy option would deliver the greatest benefit at the lowest cost to the institution. While pharmaco-economic analyses are limited to the country they are conducted in; such research is helpful to understand the cost-benefit of comparative therapies while offering reimbursement insight to similar National Health Systems. The three second-line treatment alternatives recommended by the National Cancer Center Network (NCCN) were compared to determine the best therapy for reimbursement. These treatments included: (1) increasing the imatinib dosage (to 800 mg day^−1^); (2) switching to sunitinib (50 mg day^−1^ on a 4-week on/2 week off treatment schedule); or (3) regulating symptoms with palliative care. Because GIST is associated with long-term therapy, the cost of treatment per patient was calculated over the duration of therapy to determine a total cost. Direct medical costs and reimbursed therapies associated with second-line GIST treatment were factored into cost calculations to derive an overall mean cost (MC) for each treatment. However, a Markov model was created to determine the MC of sunitinib as at the time of analysis sunitinib was not reimbursed in Mexico. Adverse event and survival rates from the pivotal sunitinib study by Motzer *et al* (2006) and the Demetri *et al* (2006) survival study were used to construct the model. Hypothetical IMSS reimbursement values were calculated to evaluate therapy cost. The reimbursement of GIST was based on the values set by the National Institute of Clinical Excellence (NICE) to determine the cost-effectiveness of sunitinib as compared to palliative therapy by utilising 5, 14, and 40% cuts of the highest NICE reimbursement level. Because increased morbidity and mortality is associated with advanced GIST, incremental cost-effectiveness ratios (ICERs), life years gained (LYG) and progression free months (PFM) were evaluated to obtain the survival gain for each therapy.

## MATERIALS AND METHODS

To estimate the cost and cost-effectiveness of second-line treatment this observational study collected imatinib and palliative care costs; created a Markov model, and conducted a sensitivity analysis. The investigation was conducted to determine the total cost an intervention would incur the IMSS per patient over the entire duration of second-line GIST treatment. Treatment costs were collected from the start of treatment until the documentation of tumour progression (Park *et al*, 2003; Watanabe *et al*, 2003). Further, patient data was investigated for a maximum of 5 years (referred to as the 5-year temporal horizon) so MC would be a true cost estimate of GIST care with a specific treatment. Cost-effectiveness measures were also an integral part of the analysis and were determined by using the ICER (see equation below). Specifically, LYG and PFM were calculated to determine the cost-effectiveness of each therapy. 
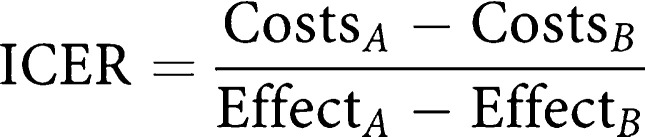


### Primary data sources: imatinib, palliative care and standard procedures

All patients (*N*=21) receiving second-line treatment for advanced GIST from 1 January 2005 to 31 December 2007 at the Hospital de Oncología were analysed to estimate the cost of care associated with imatinib, palliative care, and standard oncology procedures in Mexico. The clinical data, demographic characteristics, and medical procedures of patients receiving care for advanced GIST were obtained from patient medical charts to estimate the total cost associated with each procedure for the IMSS (see Table 1). Specifically, the mean number of visits to the oncologist, laboratory exams, radiology studies, and length of hospital stay were counted and quantified with the cost the IMSS would incur. Since data collection was dependent on medical charts, patients with insufficient information in their medical charts were excluded. Also, as the intent of this model was to characterise the MC associated in advanced GIST patients, subjects with two or more cancer types were also excluded.

Second-line therapeutic alternatives were chosen based on NCCN guidelines. Treatment alternatives evaluated in this analysis were appropriate for patients with GIST diagnosis that had non-resectable disease, widespread metastatic disease, or displayed imatinib treatment failure. To be in accordance with guidelines, the analysis estimated the cost and cost-effectiveness associated with: (1) the dosage increase of imatinib (800 mg day^−1^); (2) the use of sunitinib (50 mg day^−1^ administered for 4 weeks with 2 week rest); and (3) the administration of palliative care (National Comprehensive Cancer Network, 2006). Please note that palliative care is not always provided exclusively; instead palliative care (or rather supportive care) is often combined with imatinib or sunitinib to improve symptoms and social support. Further, since supportive care was administered with imatinib at Hospital de Oncología and is covered by the IMSS, supportive treatments are included in therapy MC estimates.

### Markov model

**Figure 1:**
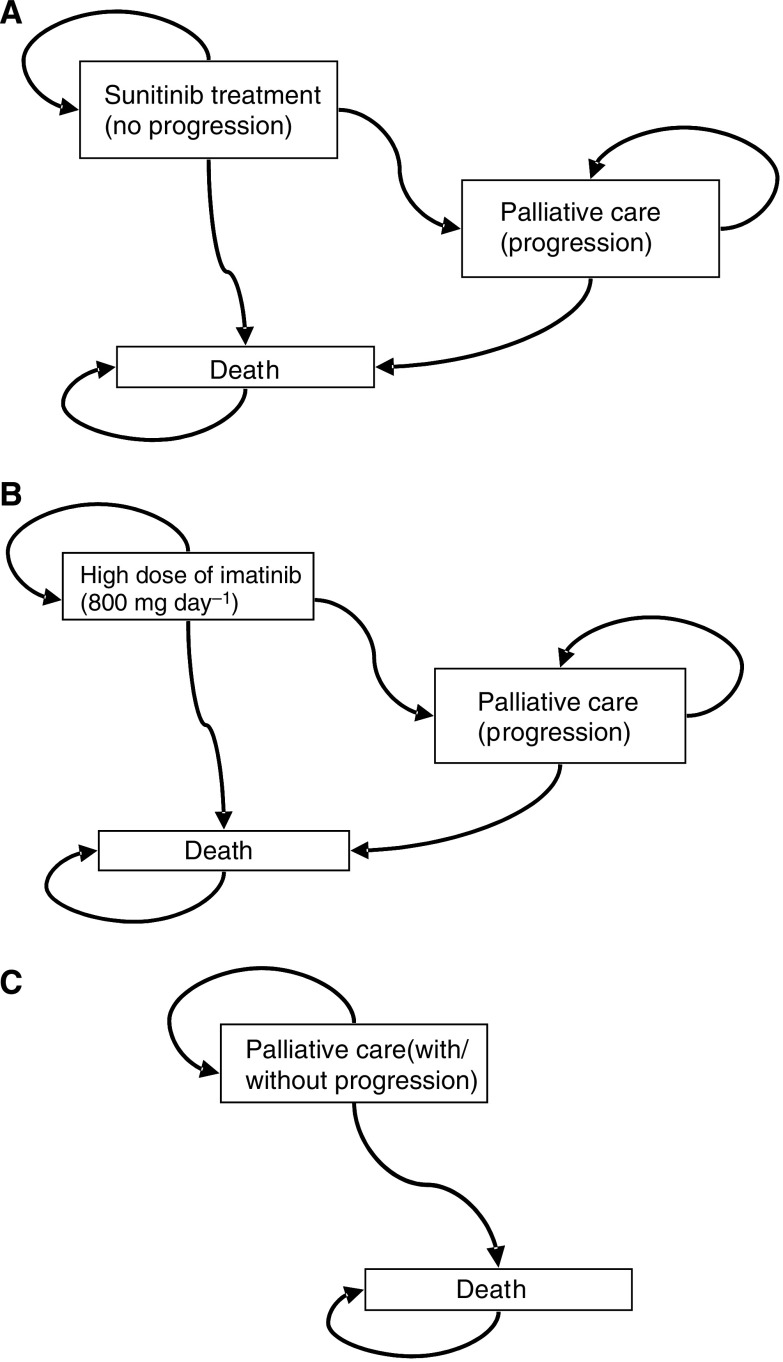
(**A**) Markov model considering sunitinib malate treatment. (**B**) Markov model considering high doses of imatinib treatment. (**C**) Markov model considering the palliative treatment.

**Figure 2:**
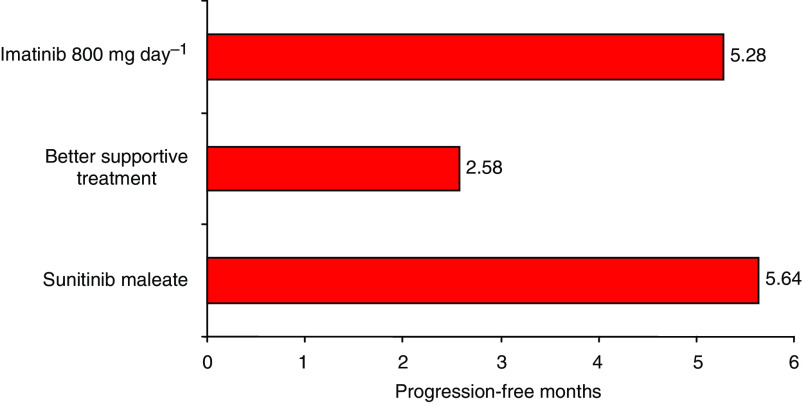
Disease progression-free months comparison, according to each therapeutic alternative, after 5 years.

**Figure 3:**
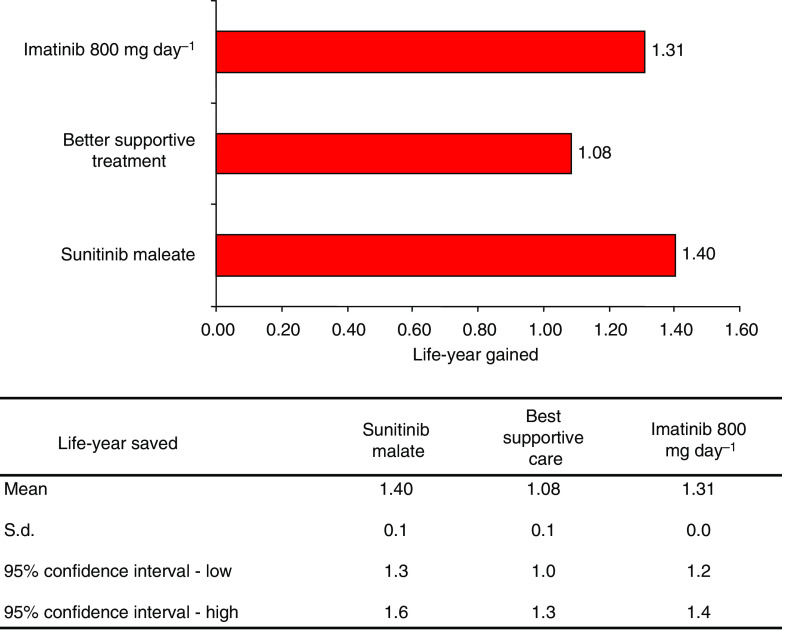
Life-year gained comparison, according to each therapeutic alternative after 5 years.

The Markov model (see Figures 1, 2 and 3) utilised results from the pivotal sunitinib phase III study by Motzer *et al* (2006) and a survival study by Demetri *et al* (2006) to ascertain the cost and cost-effectiveness associated with taking the approved 50 mg day^−1^ of sunitinib in a 4 week with 2 week rest treatment schedule. Specifically, the 54-week follow-up data of the Motzer *et al* (2006) study were extrapolated to calculate the survival time of patients taking sunitinib. As this trial was conducted in patients who had previously taken 400 mg day^−1^ of imatinib, data used in the model was representative of second-line GIST patients. Kaplan–Meier survival curves and survival tables from the Demetri *et al* (2006)study were applied to the model as this analysis followed patients to the sixth year of treatment. Six-week cyclic intervals were incorporated into the model to identify the progression of disease or any adverse events that potentially would increase or decrease sunitinib treatment cost. All costs, with the exception of sunitinib, used in the model are based on IMSS pricing and reimbursement procedures. Because sunitinib was not available in the Mexican market at the time of the analysis its cost information was provided by Pfizer Laboratories.

### Economic analysis

A 5% discount rate was applied to predict the cost and benefit of each GIST treatment (Smith and Gravelle, 2000). Application of this rate allowed a current assessment of all costs associated with a specific therapy while also capturing the cost-effectiveness of each intervention. All input prices and cost outputs were in Mexican pesos; prices were converted to US dollars (assuming 11.00 pesos per dollar exchange rate) after the analysis. Specifically, the ‘Case Mix’ methodology was used to generate cost.

### Statistical analysis

SPSS version 12.0 statistical software was used throughout the analysis. Weibull curves were estimated and then used to extrapolate the survival information over the 5-year period. The Weibull curve probability function was characterised by two parameters, lambda (*λ*) and gamma (φ), as expressed in the following formula: 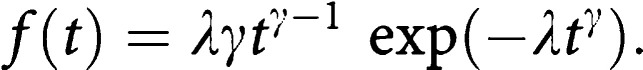 The survival function is expressed by: 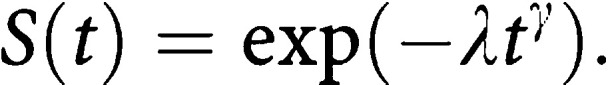 The lambda and gamma parameters, as well as s.d. and correlation coefficients were estimated (see Table 2) according to the Demetri *et al* (2006) study.

### Sensitivity analysis

The sensitivity analysis was conducted with data obtained from the Markov model. Hypothetical values were investigated at three time points during treatment, 1, 3 and 5 years specifically. In this probabilistic-type sensitivity analysis, the model was run one thousand times to evaluate robustness. Acceptability curves were also built and simulated to identify cost-effectiveness ratios for sunitinib in comparison to palliative care. As the local thresholds of cancer therapy cost have not been determined in Mexico, each curve was based on the different levels of payment, which IMSS would hypothetically contain ($27 273 USD, $36 364 USD, and $45 455 USD). Hypothetical values were based on the reimbursement of first-line treatment with imatinib set by the National Institute of Clinical Excellence (NICE) in the UK. These simulations assumed a *β* distribution for utility and a *γ* distribution for costs imputed by the model. As the *β* utility were between 0 and 1, and since *γ* values are both non negative and not normally distributed; it can be assumed that treatment costs in the model were representative of real world expenditures.

## RESULTS

### Patient demographic and clinical data

Based on information from patient medical charts, the mean patient age was 56.4 years. Thirteen patients had localised tumours in the stomach, four had localised bowel tumours, and four had a localised tumour in the rectum. Further, 14 of the 21 patients (66.7%) had metastatic liver disease and two had metastasis in the colon (Table 1).

### Medical cost data

The mean treatment follow up period of the sample was 26.2 months, during this time period the mean number of visits to the oncologist, laboratory exams, and radiology procedures were 27.0, 188.1 and 16.5 events. In addition, the mean length of hospital stay was 7.0 days per patient. Using IMSS cost data, patients treated in the sample had an annual MC per patient for medical consultations, hospitalizations, laboratory examinations, and radiology procedures of $2424.32 USD, $2657.57 USD, $566.99 USD, and $2392.67 USD, respectively. The highest annual MC per patient on drug therapy was $38 621.09 USD for a patient on imatinib. Results from the model (see Table 3) demonstrated that the highest MC (+s.d.) expected per patient over the treatment horizon was also in individuals taking high dose imatinib (800 mg day^−1^, treatment cost equivalent to $35 225.61±1253.65 USD); followed by patients taking sunitinib (50 mg day^−1^ on a 4 week on/2 week off treatment schedule, treatment cost equivalent to $17 805.87±694.83 USD); and was the lowest in patients receiving palliative care ($2071.86±472.88 USD). When the model compared the cost of sunitinib to palliative care, the ICER per patient treated with sunitinib was $15 734.23 USD.

### Cost-effectiveness analysis

**Figure 4:**
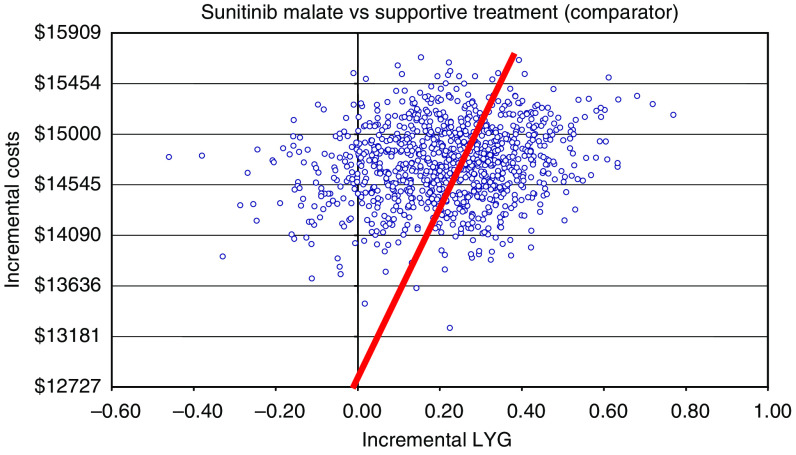
Probabilistic sensitivity analysis for sunitinib malate *vs* palliative care.

Sunitinib yielded a higher number of disease PFM than both imatinib and palliative care therapies (see Figure 4). Over the treatment horizon, sunitinib showed a mean PFM of 5.64, while imatinib and palliative care yielded smaller gains of 5.28 and 2.58 months. The incremental effectiveness of therapy as compared to palliative care was 3.1 PFM with sunitinib and 0.3 PFM with high dose imatinib.

**Figure 5:**
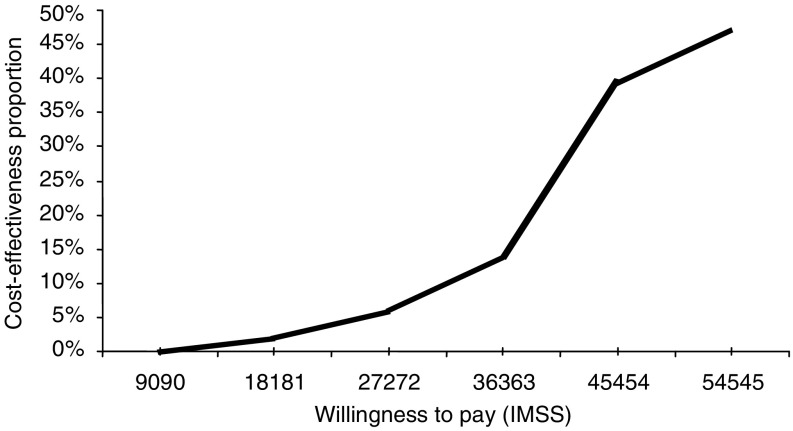
Acceptability curve for sunitinib malate *vs* palliative care (US dollars).

Patients that took sunitinib also gained more life-years than patients on other second-line treatments (Figure 5). Over the 5-year treatment horizon LYG for patients on sunitinib, imatinib and palliative care were 1.40, 1.31, and 1.08, respectively. Incrementally, sunitinib data yields a 0.32 LYG when compared to palliative treatment. Mean cost-effectiveness and ICERs among the treatment alternatives for advanced GIST over a 5-year treatment horizon are shown in Table 4.

### Sensitivity analysis

The upper threshold recommended by NICE (£25 000 or about $51 300 USD) was used for the probabilistic-type sensitivity analysis since the oncology funds available by the IMSS have yet to be determined (Wilson *et al*, 2005). A hypothetical cohort of 1000 patients taking either sunitinib or seeking palliative care was constructed to demonstrate how the cost-effectiveness of therapy changes as the cost for intervention increases. The majority of patients are located above the best fit line, and therefore the analysis predicts that the most cost-effective treatment for the majority of patients would be palliative care. Only the 38.9% of patients located below the cost threshold are predicted to receive greater cost-benefit from second-line treatment with sunitinib. However, the sensitivity analysis also predicted that the majority of patients would receive greater benefit from taking sunitinib than palliative care as patients taking sunitinib see greater increases in LYG.

Results from the sensitivity analysis were used to develop the acceptability curve for second-line treatment with sunitinib. The approximate amount of money the IMSS is willing to pay for an intervention was based on the proportion of cost-effectiveness gained by that treatment. As reimbursement for oncology compounds is unknown in Mexico, hypothetical reimbursement of the cut points in USD were $27 272.72, $36 363.63, and $45 454.54; values were derived by taking 5, 14 and 40% of the upper threshold that NICE reimburses for imatinib. The IMSS is predicted to pay more for a second-line therapy if it demonstrates a higher cost-effectiveness ratio than comparators. The cost-effectiveness of treatment with sunitinib was evaluated at 1, 3, and 5 years and the ICER was found to change the least during the first year. Patients that began treatment with palliative care saw little difference in LYG than patients on sunitinib; however, the cost difference is great. Yet, regardless of treatment type, the majority (>90%) of patients with advanced GIST die after the third year of treatment; and therefore smaller cost is incurred by the IMSS at year three regardless of therapy.

## DISCUSSION

Research has deepened scientist's understanding of the etiology of cancer and today multiple therapies exist to treat advanced GIST. Because patients and doctors are no longer reliant on palliative care for the second-line treatment of advanced GIST, it is important to determine which therapies provide the most benefit as GIST patients face serious survival and quality of life issues (Perez *et al*, 2006). Yet, GIST treatment is expensive and cost has become an important aspect of treatment as the budgets of many public medical payers are constrained. The aim of this analysis was to evaluate the cost, cost-effectiveness, and benefit associated with imatinib, sunitinib, and palliative treatment to ascertain the best second-line therapy option from the standpoint of the national health payer in Mexico, the IMSS.

It's important to underline that no randomised trial data of high dose imatinib *vs* 50 mg day^−1^ of sunitinib (in a 4 weeks on/2 weeks off treatment schedule) is available, and currently such work is just beginning. Therefore, economic modeling is a mechanism that can be used to predict trends in the absence of real data. The 800 mg day^−1^ dose of imatinib and the 50 mg day^−1^ dose of sunitinib were chosen for investigation because the medical literature has shown that: (1) patient survival improves as the imatinib dose increases (Blay *et al*, 2005); and that (2) taking sunitinib results in increased effectiveness and survival over imatinib (Demetri *et al*, 2006). PFM was calculated for all therapies over the five-year treatment duration to determine the impact of therapy on patient survival. As Figure 4 shows, PFM increases when patients receive therapy for second-line GIST; however, PFM increase is different among treatment types. Sunitinib delivered the greatest survival benefit as the regimen stopped disease progression for 5.64 months; while high dose imatinib delayed disease progression for 5.28 months; and palliative care prevented progression for 2.58 months.

Cost is another key factor to consider when evaluating GIST treatment. Patterns of resource use and medical procedures were also identified to cover the MC of GIST care. Costs associated with treatment such as hospital stay, laboratory testing, visits to the oncologist, and supportive care were calculated into the MC to capture all expenditures associated with a treatment. The most expensive treatment alternative was high dose imatinib as patients experienced an MC of $35 225.61, while the cheapest alternative was palliative care as patients had an MC of $2071.86. Alternatively, the model predicted that treatment with sunitinib would yield a median of MC of $17 805.87.

Few studies have evaluated GIST treatment and even fewer have examined possible second-line treatment alternatives from a cost-effectiveness standpoint Rubin *et al*, 2007. Case in point, the literature supports the recommendation to increase the dosage of imatinib to 800 mg day^−1^ after first-line treatment failure (Demetri *et al*, 2006) even though the elevated dosage has yet to be compared with other second-line GIST treatments. Hopefully, more therapeutic and economic comparisons will be conducted to determine the best treatment for GIST, as to date the only other economic evaluation has been conducted. This evaluation by NICE on the first-line treatment of imatinib found it to be a cost-effective GIST treatment. Since no data is available to determine the reimbursement of cancer therapies by the IMSS, the upper reimbursement threshold recommended by NICE (£25 000 or about $51 300 USD) was used to conduct the sensitivity analysis. When sunitinib was compared to palliative care, only 38.9% of patients are expected to receive greater cost-benefit from sunitinib. Even though the sensitivity analysis showed high variability from the actual ICER; the results obtained from the model support, the inclusion of sunitinib as a reimbursable therapy to manage GIST. Because GIST patients tend to have lower life expectancies, the model favours cheaper palliative care as the best solution. It is difficult to put a price on life, therefore the effectiveness and benefit measures should be given more weight in GIST studies Badalamenti *et al*, 2007. As Figure 4 shows, the majority of patients would experience greater LYG on sunitinib than on palliative care. Further, when LYG was compared among therapies the model predicted that patients taking sunitinib would experience greater LYG than patients taking imatinib or palliative care (see Figure 5). Acceptability curves for sunitinib were constructed to predict the reimbursement of GIST treatment by the IMSS. As the curves showed low percentages with different cut-points for willingness to pay (Figure 5), alleviating unnecessary costs to save on overall treatment will be key.

In closing, the weaknesses of the analysis should be discussed. First, the generalizability of the study is compromised as a mathematical model was used to predict the cost, cost-effectiveness, and benefit for the three treatment alternatives (imatinib, sunitinib and palliative care). Different outcomes occur in subjects that undergo the same procedure, and unfortunately a model cannot capture or predict all results. Second, as GIST is a rare disease and current treatment had to be obtained from a sample of 21 mostly female patients; it is possible that imatinib and palliative care data may not be representative of all patients on second-line GIST treatment in Mexico. However, as all eligible patients were analysed and as GIST has no sex-specific implications, the sample should be representative. Nonetheless, in the future we hope to validate the model and amend these issues by using real patient data and a large patient database. Lastly, this analysis cannot predict how cost and benefits would change if the dose or administration of sunitinib changed; therefore the analysis is limited to describing the reimbursement, cost, and benefits of patients taking the 37.5 mg day^−1^ dose of sunitinib in a 4 week on/2 week off treatment schedule.

## CONCLUSION

The model predicts that 50 mg day^−1^ of sunitinib administered in a 4-week with 2 week rest treatment schedule would be the most cost-effective option in second-line treatment. Therefore, the IMSS would not only observe major savings by reimbursing sunitinib, but also would provide patients with access to a new therapy with the greatest survival benefits in second-line GIST.

## Figures and Tables

**Table 1:** Characteristics of 21 advanced gastrointestinal stromal tumor (GIST) patients who were treated at the Hospital de Oncología, IMSS

	**Patient data**
**Characteristic**	***n*=21**
*Age (years)*	
Mean (s.d.)	56.4 (12.9)
	
*Sex, no* (%)	
Female	17 (81)
Male	4 (19)
	
*Tumour localisation, n* (%)	
Stomach	13 (16.9)
Small bowel	4 (19.0)
Rectum	4 (19.0)
	
*Metastatic disease, n* (%)	
Liver	14 (66.7)
Colon	2 (9.5)
	
*Follow-up period (months)*	
Mean (s.d.)	26.19 (20.9)
Range	6–73
	
*No of oncologist visits per patient*	
Mean (s.d.)	27.0 (15.2)
Range	9–68
	
*No of laboratory exams per patient*	
Mean (s.d.)	188.0 (13.71)
	
*No of radiology studies per patient*	
Mean (s.d.)	16.46 (4.0)
	
*Hospital length stay per patient (days)*	
Mean (s.d.)	7.0 (9.5)

**Table 2:** Parameters used to build survival Weibull curves

	**Lambda^1^**	**Gamma^1^**	**Correlation coefficients**	***R*^2^ 2**
Total survival: best supportive care	0.013641 (0.002600)	1.06418 (0.06225)	−0.98360	0.94424
Total survival: sunitinib^3^	0.002183 (0.000551)	1.40080 (0.07333)	−0.9940	0.96441
Free progression survival: best supportive care	0.039030 (0.009569)	1.49029 (0.11946)	−0.9803	0.92923
Free progression survival: sunitinib	0.042038 (0.006473)	0.94287 (0.05051)	−0.9774	0.93136

1 Mean and s.d.

2 R^2^ describes the model explanation capacity with the original data (e.g. when the estimation is closer to 1.0, the survival curves have a better explanation capacity).

3 The utility levels for each stratum are the same for high doses of imatinib.

**Table 3:** Total costs expected per patient (US dollars, 2006)

**Costs**	**Sunitinib**	**Best supportive care**	**High doses imatinib**
Mean	$17 805.87	$2071.86	$35 225.61
s.d.	$694.83	$472.88	$1253.65
Low 95% confidence interval	$15 377.23	$1543.32	$31 381.21
High 95% confidence interval	$19 815.68	$2869.36	$38 705.18

**Table 4:** Cost-effectiveness (mean and incremental) with the three treatment alternatives, 5-year horizon

	**CER**	**CER**	**ICER**	**ICER**
**Alternative**	**Years of free survival progression**	**Years gained**	**Years of free survival progression**	**Years gained**
Sunitinib	35 057.22	11 862.35	—	—
Palliative care	8869.06	1754.80	56 612.55	46 108.89
High doses imatinib	84 540.50	28 423.57	Dominated	Dominated

CER: Cost-effectiveness ratio (mean); ICER: Incremental cost-effectiveness ratio.
